# fNIRS Responses in Professional Violinists While Playing Duets: Evidence for Distinct Leader and Follower Roles at the Brain Level

**DOI:** 10.3389/fpsyg.2019.00164

**Published:** 2019-02-05

**Authors:** Patricia Vanzella, Joana B. Balardin, Rogério A. Furucho, Guilherme Augusto Zimeo Morais, Thenille Braun Janzen, Daniela Sammler, João R. Sato

**Affiliations:** ^1^Núcleo Interdisciplinar de Neurociência Aplicada, Universidade Federal do ABC, Santo André, Brazil; ^2^Centro de Matemática, Computação e Cognição, Universidade Federal do ABC, São Bernardo do Campo, Brazil; ^3^Hospital Albert Einstein, Instituto do Cérebro – Instituto Israelita de Ensino e Pesquisa Albert Einstein, São Paulo, Brazil; ^4^NIRx Medizintechnik GmbH, Berlin, Germany; ^5^Faculty of Music, University of Toronto, Toronto, ON, Canada; ^6^Max Planck Institute for Human Cognitive and Brain Sciences, Leipzig, Germany

**Keywords:** joint action, musical ensemble performance, naturalistic paradigm, leadership, hyperscanning, fNIRS

## Abstract

Music played in ensembles is a naturalistic model to study joint action and leader-follower relationships. Recently, the investigation of the brain underpinnings of joint musical actions has gained attention; however, the cerebral correlates underlying the roles of leader and follower in music performance remain elusive. The present study addressed this question by simultaneously measuring the hemodynamic correlates of functional neural activity elicited during naturalistic violin duet performance using fNIRS. Findings revealed distinct patterns of functional brain activation when musicians played the Violin 2 (follower) than the Violin 1 part (leader) in duets, both compared to solo performance. More specifically, results indicated that musicians playing the Violin 2 part had greater oxy-Hb activation in temporo-parietal (*p* = 0.02) and somatomotor (*p* = 0.04) regions during the duo condition in relation to the solo. On the other hand, there were no significant differences in the activation of these areas between duo/solo conditions during the execution of the Violin 1 part (*p*’s > 0.05). These findings suggest that ensemble cohesion during a musical performance may impose particular demands when musicians play the follower position, especially in brain areas associated with the processing of dynamic social information and motor simulation. This study is the first to use fNIRS hyperscanning technology to simultaneously measure the brain activity of two musicians during naturalistic music ensemble performance, opening new avenues for the investigation of brain correlates underlying joint musical actions with multiple subjects in a naturalistic environment.

## Introduction

Musical ensemble performance is a highly sophisticated activity involving complex motor, cognitive, and social processes (Keller et al., 2014; D’Ausilio et al., 2015; Volpe et al., 2016). Ensemble musicians must precisely coordinate their actions with those of their partners in the order of hundreds of milliseconds to produce a consistent music performance. Temporally precise interpersonal coordination requires cognitive-motor skills that enable musicians “to represent joint action goals and to anticipate, attend, and adapt to others’ actions in real-time” (Sebanz et al., 2006; Sebanz and Knoblich, 2009; Keller et al., 2014; see also: Novembre and Keller, 2014). Ensemble cohesion is also determined by the social dynamics between performing musicians and the differential roles that partners can play during joint actions (D’Ausilio et al., 2015; Volpe et al., 2016). Studies demonstrated, for instance, that ensemble leaders use auditory information as well as cueing gestures, such as head movements and body sway, to facilitate interpersonal coordination and communicate beat position and expressive intentions, indicating that musicians employ a number of non-verbal communication strategies to improve ensemble cohesion (Goebl and Palmer, 2009; Chang et al., 2017; Bishop and Goebl, 2018).

The recent development of neuroimaging techniques such as hyperscanning, which allows the simultaneous recording of brain activity of multiple subjects, has greatly expanded our understanding of the neurobiological mechanisms underlying joint actions (for review: Montague et al., 2002; Bekkering et al., 2009; Scholkmann et al., 2013; Babiloni and Astolfi, 2014). Several recent electroencephalography (EEG) studies have investigated the neural basis of joint action in music performance using hyperscanning methods (Lindenberger et al., 2009; Konvalinka et al., 2010; Babiloni et al., 2011, 2012; Sänger et al., 2012; Loehr et al., 2013; Müller et al., 2013; for review: Acquadro et al., 2016). Overall, findings suggest that inter-brain synchronization is crucial in tasks that require coordination of one’s own and their partner’s actions in real time. Specifically, studies examining intra- and interbrain synchronization between musicians playing in duet reported that moments that put particularly high demands on musical coordination increase phase locking within and between the musicians’ brains (Sänger et al., 2012). Furthermore, it was shown that the leaders of these duets had an increased phase locking in the delta frequency range already before coordinated playing onset, whereas phase locking started only after the play onset in followers, which indicates that the musical roles of leader and follower are associated with differences in brain synchronization patterns (Sänger et al., 2012, 2013). Konvalinka et al. (2014) used dual-EEG to measure neural activity during a joint finger-tapping task, and demonstrated that interactive situations were associated with stronger suppression of alpha and low-beta oscillations over motor and frontal areas of the brain, and also, that leaders (those who adapted less to their partners) had notably stronger alpha suppression in frontal regions, possibly indicating an increase in internally driven or self-oriented decision-making processes.

The brain correlates of the leader-follower dynamics in joint actions have been recently explored in two functional magnetic resonance imaging (fMRI) studies (Fairhurst et al., 2014; Chauvigné et al., 2018). Fairhurst et al. (2014) investigated the mechanisms of how individuals lead using an auditory finger-tapping task where participants tapped in coordination with a virtual partner. In Chauvigné et al. (2018), trained couple dancers engaged in bimanual dance-related movements while one of the dancers had the brain activity recorded and the other was standing next to the bore of the magnet. Collectively, these studies concur with the notion that leadership in a joint task is associated with more internally driven motor processes as shown by a greater activation in brain regions involved in self-initiated actions, motor planning, decision making, and spatial orientation, including the supplementary motor area, dorsal anterior cingulate cortex, cingulate motor area, premotor cortex, right inferior frontal gyrus, and superior parietal lobule. On the other hand, following seems more associated to sensory and externally oriented processing patterns as revealed by a greater activation in areas involved in somatosensation, proprioception, motion tracking, and social perception, such as the primary and secondary somatosensory cortices, the motion area of the middle temporal region (MT+/V5), and posterior superior temporal sulcus (Chauvigné et al., 2018). These findings, therefore, highlight the complementary feature of leading and following, with leadership being more associated to motor-related self-initiation processes and following being more related to the processing of external sensory information (Fairhurst et al., 2014; Chauvigné et al., 2018). Interestingly, Chauvigné et al. (2018) also reported that in the interactive task (where partners had a more symmetrical role) there was increased activity in areas associated with the mentalizing network, including the posterior superior temporal sulcus, temporo-parietal junction (TPJ), and medial prefrontal cortex. The mentalizing network (or theory of mind network) has been consistently reported in interactive tasks and empathetic processes, and it is thought to be involved in the ability to understand others’ mental states, to predict their intentions, and to consciously alter one’s behavior accordingly (Gallagher and Frith, 2003; Van Overwalle, 2009; Carter and Huettel, 2013; Schurz et al., 2014; Krall et al., 2015; Yang et al., 2015).

Although fMRI studies have considerably advanced our understanding of the brain networks involved in social interactions, this technology does not favor the investigation of joint actions in a naturalistic context. For this reason, a growing number of studies have been resorting to functional near-infrared spectroscopy (fNIRS) for the functional assessment of the brain. Similarly to fMRI, fNIRS indirectly infers neural activity through the evaluation of hemodynamics and local oxygenation of the cortical surface. This technique uses low-energy light receptors and emitters to measure absorption in surface tissues of the human brain, with an average spatial penetration resolution on the order of 5–10 mm. In this manner, it is possible to analyze local alterations in the concentrations of oxygenated and deoxygenated hemoglobin as a response to functional brain activities (Villringer et al., 1993). fNIRS also has important advantages in relation to its portability, lower sensibility to movement artifacts, reduced cost, and the possibility to evaluate changes in the concentration of non-oxygenated hemoglobin. Indeed, fNIRS has been successfully implemented as a non-invasive method to study brain function, especially in naturalistic experimental paradigms that aim to assess the hemodynamic correlates during the execution of tasks in ecological conditions (Cui et al., 2012; Scholkmann et al., 2013; Babiloni and Astolfi, 2014; Balardin et al., 2017). It has also been previously used to study cortical hemodynamics during music perception (Santosa et al., 2014).

The present study aims at investigating the brain correlates underlying leader-follower relationships in musical ensemble performance in a naturalistic paradigm using fNIRS. Differently from recent EEG studies exploring the inter-brain synchronization of brain oscillations in musical ensembles, here we focused on identifying differences in the recruitment of specific cortical regions depending on task manipulation. For that, we simultaneously measured the brain activity of pairs of violinists playing a piece of classical music in duet or solo. The music piece was chosen to naturally induce leader (First Violin) and follower roles (Second Violin) without the need to explicitly pre-assign these roles. This way, we could assess how the differential roles that partners can play during joint actions would develop in a naturalistic setting and whether these distinct roles would be evident at the brain level. Based on recent findings, we focused our analyses on sensorimotor and temporo-parietal areas and hypothesized that there would be greater activation in these regions during the duo condition in comparison to solo. We also expected to find greater activation in these areas in the violinist that takes on the role of the follower during the duet than in the leader of the duo because of greater demands on sensorimotor coordination and motor prediction.

## Materials and Methods

### Participants

We collected data from ten violinists (five duets). The mean age of the participants was 32.7 years (*SD* = 8), all were right-handed, and half of them were women. To ensure a similar level of musical expertise and technical mastery of the instrument, the musicians recruited in this study were required to have a minimum of 8 years of uninterrupted violin training. Our final sample included musicians with an average of 15 years (*SD* = 4) of uninterrupted training and an average of musical training onset of 9.3 years (*SD* = 3.9). They were all professional musicians regularly performing in orchestras and/or chamber music ensembles in the Greater Sao Paulo area. The experimental procedures conformed with the Declaration of Helsinki and were approved by the Research Ethics Board of the Universidade Federal do ABC. All participants were fully informed about the nature of the study and provided written informed consent.

### Musical Material

The musical piece used in this study was the Duo n^o^ 37 (Prelude and Canon) – which is one of the 44 Duos for Violins written in 1931 by the Hungarian composer Béla Bartók (1881–1945). These duos are short pieces based on a repertoire of popular songs and dances of various origins (Hungarian, Romanian, Serbian, Slovak, Slavic, and Arabic), to which Bártok added his own distinguishing contours. Duo n^o^ 37 places similar technical and expressive demands on both violinists, which makes this piece ideal for studying coordination, synchronization, and empathetic interactions between members of an ensemble during the performance. Even though the selected piece of music presented similar technical and expressive demands for both musicians, some aspects of its structure (e.g., beginning of phrases, more rhythmic activity in places with marked tempo changes) could lead the violinist playing the First Violin to naturally assume a leadership position throughout most of the performance.

### Procedure

One week prior to the experiment session, participants were sent the music score of Bartók’s Duo n^o^ 37 (Universal Edition) and were instructed to study both parts (Violins 1 and 2) as if they were to perform it in a concert in the following week. On the day of the experiment, prior to the performance of the piece, detailed below in the section describing the fNIRS task, participants had a cap with the optodes of the NIRS equipment placed on their scalps. At the end of the session, participants completed a short demographic survey answering questions related to lateral dominance, musical training, and musical experience, as well as on their degree of comfort or discomfort throughout the data acquisition process using NIRS. In this questionnaire, violinists also rated on a continuous scale the length of time they felt like the leader or follower while performing the First and Second Violin parts, the level of understanding with his/her partner while playing, and their level of satisfaction with the duo’s performance overall. These ratings were converted into numerical 0–12 scores, and differences between runs were evaluated using a paired samples *t*-test with significance level set at 5%.

#### fNIRS Task

Each pair of musicians were scanned in one session which was divided into two identical runs, except that the violinists performed a different part in the second run (the violinist that had performed Violin 2 in the first run performed Violin 1 in the second run, and vice-versa).

**Figure 1:**
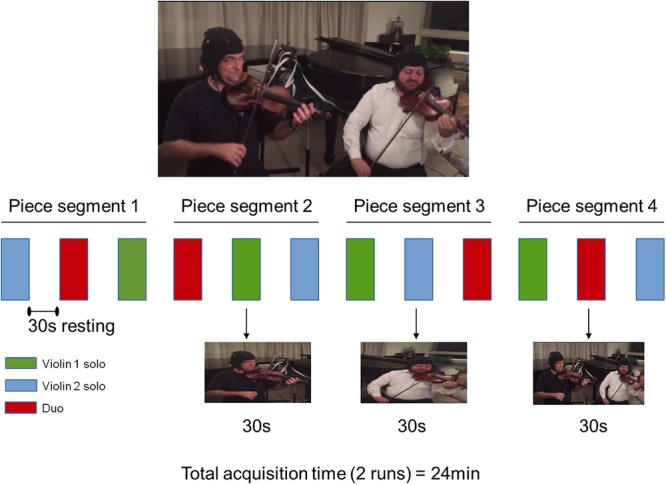
Experimental procedure in one run. Four segments of the musical piece were split in three parts played as Violin 1 solo (green), Violin 2 solo (blue), and Violins 1 and 2 in duo (red). The order of performance of each violin part was counterbalanced across segments. Violin 1 and Violin 2 roles were reversed in the second run (not shown). Written informed consent was obtained from the violinists for publication of this image.

The music was divided into four segments, indicated in the score, each with an approximate duration of 30 s. In each run of data acquisition, musicians were instructed through a recording to perform each of the four music segments under two different conditions: playing alone the Violin 1 or Violin 2 parts (solo condition) and playing together (duo condition). These active conditions were interweaved with blocks of resting baseline of the same duration. The onset of each block was indicated by an audio recording. Violinists were seated next to each other facing their music scores. The sequence of the active conditions was counterbalanced within musical segments and between runs. An illustration of one run of the experimental paradigm is provided in Figure 1.

#### fNIRS Data Acquisition

The hemodynamic signals were obtained from the optical changes collected using a continuous wave, fNIRS system (NIRScout, NIRx Medical Technologies, Glen Head, NY) with 16 LED illumination sources emitting two wavelengths of near-infrared light (760 and 850 nm) and 16 optical detectors. The optodes were shared between the two participants, thus providing a simultaneous acquisition. Signals were recorded at a sampling rate of 7.81 Hz.

**Figure 2:**
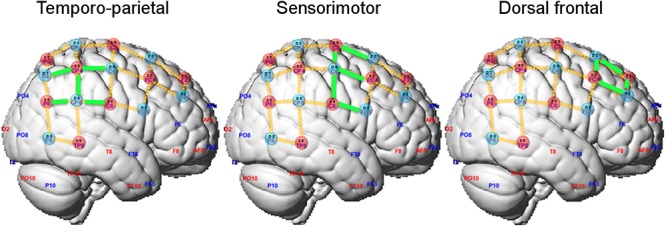
Optode layout and definition of regions of interest (ROIs). Selected channels in each ROI are marked in green.

Sources and detectors were positioned on the measuring cap with reference to the 10–10 international system (Sharbrough et al., 1991). The optodes’ spatial distribution on the cap was chosen to result on channels (i.e., source-detector pairs) with standard inter-optode distances of approximately 30 mm. Optodes placement was restricted to the right hemisphere not to constrain the position of the head of the violinist on the instrument. In total, 23 channels covered regions of the motor and sensorimotor cortices as well as the temporoparietal junction (Figure 2 and Supplementary Figure S1).

### fNIRS Data Analysis

The raw intensity data were analyzed offline using the NIRS Toolbox software (Santosa et al., 2018). The optical signals of each channel were converted to oxygenated hemoglobin (oxy-Hb) and deoxygenated hemoglobin (deoxy-Hb) concentration changes by using the modified Beer-Lambert equation (Delpy and Cope, 1997) using a differential pathlength factor of 6 for both wavelengths (Strangman et al., 2013). This toolbox uses an analytical approach in which physiological noise and motion artifacts are dealt with statistically within the general linear model (Huppert, 2016). Statistical analysis was carried out using an autoregressively whitened robust regression model (Barker et al., 2013). In brief, a general linear model (GLM) was applied to the fNIRS time-series with three regressors of interest, which modeled the 30s duration of each experimental condition (i.e., playing solo as Violin 1, playing solo as Violin 2, and playing together as a duo). These regressors were convolved with a time-to-peak canonical hemodynamic response function of 4s. An iterative auto-regressive (AR) filtering was used to eliminate the serial correlations in the residual of the model by employing a linear whitening filter (*S^-1^*) on both sides of the equation (e.g., *S^-1^⋅y = S^-1^⋅X*β *+ S^-1^⋅ε*). This AR model is estimated by Bayesian information criteria (BIC) search of AR model order to whiten the residual of the linear regression model. The solution to this model is then iteratively estimated by robust regression.

We adopted a region-of-interest (ROI) analysis approach and defined two ROIs according to our *a priori* hypotheses on the possible different recruitment of cortical regions in playing in ensemble compared to playing solo. The sensorimotor ROI encompassed channels 2-4, 3-2, 3-4, 4-3, 4-4, and the temporo-parietal ROI channels 3-6, 4-6, 5-4, and 5-7 (highlighted in green in Figure 2). Additionally, we defined a dorsal frontal ROI as control region composed of the remaining channels (channels 1-1, 1-2, 2-1, and 2-2). No differences between leaders and followers were expected in this ROI. For each ROI, the contrast of beta estimates between playing in duo and solo relative to the resting fixation baseline were averaged by participant across the relevant channels, and its median was exported for further analysis in the R platform for Statistical Computing^1^. Duo and solo conditions were then statistically compared, separately for each ROI and for playing Violin 1 or Violin 2, using paired Wilcoxon non-parametric tests (two-tailed). To assess whether effects differed between Violin 1 or 2, the differential effects (duo-minus-solo) were compared between Violins using Wilcoxon tests. The rate of accepted false positive results was set at 5% (corrected for multiple comparisons). Statistical analysis was conducted on changes in both oxy-Hb and deoxy-Hb, but the latter did not reach significance, and its results are not reported.

## Results

**Figure 3:**
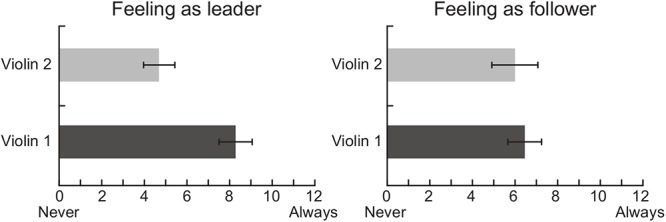
Self-reports of musicians’ perception regarding the amount of time perceived as being the leader and the follower of the duo while playing Violin 1 or Violin 2 parts.

Data analysis of the self-report questionnaires indicated that the amount of time musicians perceived as being the leader of the duo was lower when they played the Violin 2 part than the Violin 1 part [*t*(9) = 2.59, *p* = 0.029, Figure 3]. No statistical difference was found for the time perceived as being follower [*t*(9) = 0.29, *p* = 0.778]. Musicians presented high ratings on the level of understanding with his/her partner in the duo condition (Violin 1: *M* = 9.8 *SD* = 2.81; Violin 2: *M* = 9.8 *SD* = 2.55) and also indicated high levels of satisfaction with the duo’s performance (Violin 1: *M* = 9.32 *SD* = 2.14; Violin 2: *M* = 9.07 *SD* = 2.65).

**Figure 4:**
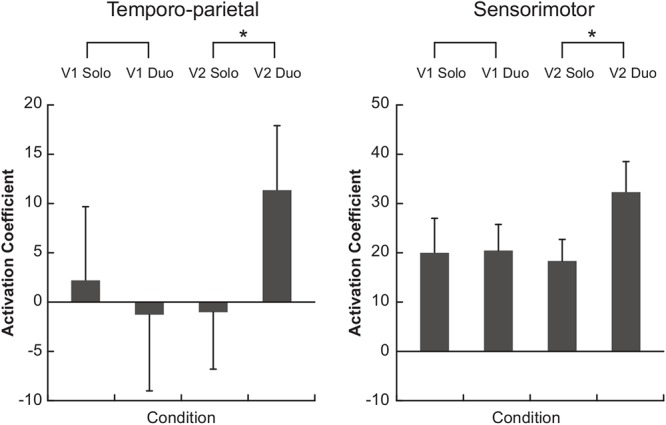
Functional near-infrared spectroscopy (fNIRS) results. Bars represent the median of the activation coefficients (beta) across subjects (±1 SEM). V1, violin 1 part; V2, violin 2 part. ^∗^*p* < 0.05.

Comparisons of the hemodynamic signals during the duo compared to the solo condition showed a greater oxy-Hb activation in the temporo-parietal ROI when musicians performed the Violin 2 part (*p* = 0.020), but not when they played the Violin 1 part (*p* = 0.232) (Figure 4, left panel). A similar result was observed for the sensorimotor ROI, in which oxy-Hb levels were also higher in duo than solo when musicians played the Violin 2 part (*p* = 0.048), but not the Violin 1 part (*p* = 0.846) (Figure 4, right panel). These differences were visible in the majority of the violinists at individual level (see Supplementary Figure S2), and were significantly stronger in Violin 2 than Violin 1 as confirmed by a statistical comparison of the difference values (duo-minus-solo) between Violins (temporo-parietal: *p* = 0.0195; sensorimotor: *p* = 0.027). No difference in oxy-Hb was found in duo vs. solo in the dorsal frontal control ROI, neither when musicians performed the Violin 1 (*p* = 0.780) nor the Violin 2 part (*p* = 0.232; no difference between Violins: *p* = 0.375).

## Discussion

The present study investigated the brain correlates of musical ensemble performance by comparing the functional neural activity measured during a naturalistic violin duo performance to brain activity recorded during solo performance. We hypothesized that the duo condition would elicit greater activation in sensorimotor and temporo-parietal areas compared to solo and that these regions would be more active in musicians taking on the follower role in the duo. Our results confirmed these hypotheses, as somatomotor and temporo-parietal regions, but not a dorsal frontal control region, showed greater oxy-Hb response during the ensemble performance in the violinist playing the Violin 2 part.

The self-reports indicated that, during the duo condition, violinists playing Violin 1 perceived themselves more as the leaders of the duo than those playing Violin 2. These roles were established without explicit instruction to do so. It is possible that the leader-follower arrangement of classical ensembles was implicitly maintained during the duo performance given that the violinists who participated in this study were all professional musicians with classical training. This observation could be related to the fact that the First Violin is conventionally associated with a leadership role in classical ensembles, often having the responsibility of coordinating ensemble cohesion and facilitating interpersonal coordination during a performance (Timmers et al., 2014; Wing et al., 2014; Chang et al., 2017; Bishop and Goebl, 2018), but also taking on administrative roles in larger groups such as orchestras (Murnighan and Conlon, 1991). Alternatively, the music piece selected for this study may have also influenced how the roles were intuitively assigned. Although the music performed (Bartók’s Duo n^o^ 37) presents similar technical and expressive demands for both musicians, some aspects of its structure (e.g., beginning of phrases and rhythmic patterns) may lead the musician playing the Violin 1, rather than Violin 2, to naturally assume a leadership position during performance. It is interesting to note that, while musicians seemed more aware of being in the leader position when playing the First Violin, both players indicated to similarly, follow the other, i.e., to adapt to one another in an ongoing and mutual manner (right panel in Figure 3). This finding concurs with previous research suggesting that leader and follower roles are not necessarily dichotomous positions, but depend on the degree to which each partner adapts to others when cooperating to achieve a shared goal (Goebl and Palmer, 2009; Pecenka and Keller, 2011; Fairhurst et al., 2014; Wing et al., 2014; Chauvigné et al., 2018).

Analysis of the hemodynamic fNIRS signals showed that musicians playing the Violin 2 part had greater oxy-Hb activation in right temporo-parietal and sensorimotor regions during the duo compared to the solo condition. No such differences were found in Violin 1 players. Increased temporo-parietal activity when playing Violin 2 in a duet is in line with the involvement of temporo-parietal regions in joint action and social interactions (for review: Sebanz et al., 2006; Kokal et al., 2009; Yang et al., 2015). More specifically, TPJ is part of the mentalizing network supporting theory of mind (Gallagher and Frith, 2003; Kokal et al., 2009; Carter and Huettel, 2013; Schurz et al., 2014; Yang et al., 2015) and higher-order processing of social behaviors (Decety and Grèzes, 2006; Carter and Huettel, 2013; Krall et al., 2015), including the perception of agency (Newman-Norlund et al., 2008; Tsakiris et al., 2008) and inference of others’ intentions and goals (Van Overwalle, 2009). These processes may be taxed particularly strongly in Violin 2 players who more naturally assumed a follower rather than a leadership role in the duets (other than Violin 1 players). Likewise, posterior superior temporal sulcus (pSTS) known to process dynamic social stimuli (for review: Yang et al., 2015), biological motion (Grossman et al., 2005; Pelphrey et al., 2005; Saygin, 2007; Gilaie-Dotan et al., 2013; Van Kemenade et al., 2014; Vetter et al., 2015), and facial expressions (Bernstein and Yovel, 2015), often by integrating multisensory information (Beauchamp et al., 2004, 2008), may have been more strongly involved in Violin 2 players in their assumed role as followers.

Along the same lines, the increased activity in sensorimotor areas in musicians playing Violin 2 may reflect greater demands on brain networks involved in motor simulation and self/other coordination in real time joint actions. Our findings concur with studies showing that rhythmic interpersonal coordination in joint musical actions relies on one’s ability to simulate and anticipate the partner’s actions (Sebanz et al., 2006; Sebanz and Knoblich, 2009; Keller et al., 2014; Novembre and Keller, 2014) and to co-represent the co-performer’s action in one’s own motor system (Calvo-Merino et al., 2005, 2006; Schütz-Bosbach et al., 2006; Knoblich and Sebanz, 2008; Rizzolatti and Sinigaglia, 2010) strengthened by motor familiarity with the partner’s part (as was the case in the present study; Novembre et al., 2012, 2014; Loehr et al., 2013; Ragert et al., 2013). Most importantly, however, recent findings suggest that followers prioritize the task of synchronizing with their partners and maintaining cohesion, while leaders focus more on stabilizing the tempo of their own performance (Fairhurst et al., 2014). Hence, Violin 2 (but not Violin 1) players may have relied more heavily on motor simulation as a mechanism to predict the other player’s action in real time based on their own action repertoire (Sebanz et al., 2006; Sebanz and Knoblich, 2009; Van Der Steen et al., 2015) to maintain ensemble cohesion during joint musical performance.

Together, our results suggest that interpersonal coordination during ensemble musical performance relies on one’s ability to understand and predict the partner’s actions and mental states, engaging a range of brain areas involved in processing dynamic social information. However, why were effects observed only in Violin 2, not in Violin 1 players? This finding seems to differ from the results of Fairhurst et al. (2014) and Chauvigné et al. (2018), who reported greater activity, for example, in sensorimotor areas when perceiving greater influence on a virtural partner (Fairhurst et al., 2014), and when imposing particular ‘leadership’ demands on participants, such as the improvisation of new dance-related movements (Chauvigné et al., 2018). Both studies attributed the enhanced sensorimotor activity to the self-initiation of actions, motor planning, and navigation, that is, self-oriented processes particularly relevant for leaders, but also required in solo performance. In other words, it is possible that these processes did not differ significantly during the duo and solo performance of the Violin 1 part in the present study.

The combined findings have significant implications for the field of joint action and music performance research by demonstrating the feasibility to monitor brain hemodynamic changes during naturalistic and unconstrained music ensemble performance using fNIRS. This technology thus complements well-known neuroimaging techniques and may be extended to tasks involving more than two co-performers interacting freely in naturalistic environments. However, a few limitations to this study need to be acknowledged, including the small number of participants and the acquisition of fNIRS data from the right hemisphere only. Optode placement on the left hemisphere would have hindered free movement and conflicted with the position of the violin. Further research with other ensemble arrangements is needed to confirm the present findings and assess whether there may be hemispheric differences in the activity of temporo-parietal and somatomotor regions relating to the leader and follower roles in music performance. Future studies may benefit from using accelerometers to objectively estimate the contribution of head and body movements to the observed results, and from registering fNIRS data to anatomical MRI scans to better describe the correspondence between the studied ROIs and their underlying anatomy. Finally, for a better understanding of the origins of the observed task-related changes in oxy-Hb, but not in deoxy-Hb, we suggest to simultaneously monitor systemic variables using, for example, short-distance channels and/or breath and heart rate monitoring.

## Conclusion

The present study used a novel fNIRS hyperscanning approach in natural violin duets to investigate the brain correlates underlying leader/follower roles. Musicians playing the Violin 2 who intuitively assumed a follower role had greater oxy-Hb activation in temporo-parietal and somatomotor regions when performing the duo condition compared to solo. No such differences were found in those playing the Violin 1 who perceived themselves as leaders. These findings suggest that ensemble cohesion during a musical performance may impose particular demands on musicians in the follower role, especially in brain areas associated with the processing of dynamic social information and motor simulation. To our knowledge, this work represents the first use of a single NIRS instrument for simultaneous measurements of brain activity during a naturalistic music ensemble performance, opening new avenues for further investigation of brain correlates underlying joint musical actions with multiple subjects in a naturalistic environment.

## Author Contributions

PV, JS, and JB conceived and designed the study. PV, JB, and RF acquired the data. GZ and JS analyzed the statistics. PV, JB, JS, TBJ, and DS interpreted the data and prepared the manuscript.

## Conflict of Interest Statement

GZ is employed by NIRx Medizintechnik GmbH. The remaining authors declare that the research was conducted in the absence of any commercial or financial relationships that could be construed as a potential conflict of interest.

## Footnotes

1 www.r-project.org

## Supplementary Material

Figure S1 Optode setup overlaid onto the 10–20 international system. Sources are depicted in red, detectors in blue, and channels in green.

Figure S2 Individual beta values of the group comparison between duo versus solo when playing as Violin 1 and Violin 2.

## References

[B1] Acquadro M. A. S. Congedo M. De Riddeer D. (2016). Music performance as an experimental approach to hyperscanning studies. *Front. Hum. Neurosci.* 10:242. 10.3389/fnhum.2016.00242 27252641 PMC4879135

[B2] Babiloni C. Buffo P. Vecchio F. Marzano N. Del Percio C. Spada D. (2012). Brains “in concert”: frontal oscillatory alpha rhythms and empathy in professional musicians. *Neuroimage* 60 105–116. 10.1016/j.neuroimage.2011.12.008 22186679

[B3] Babiloni C. Vecchio F. Infarinato F. Buffo P. Marzano N. Spada D. (2011). Simultaneous recording of electroencephalographic data in musicians playing in ensemble. *Cortex* 47 1082–1090. 10.1016/j.cortex.2011.05.006 21664610

[B4] Babiloni F. Astolfi L. (2014). Social neuroscience and hyperscanning techniques: past, present and future. *Neurosci. Biobehav. Rev.* 44 76–93. 10.1016/j.neubiorev.2012.07.006 22917915 PMC3522775

[B5] Balardin J. B. Zimeo Morais G. A. Furucho R. A. Trambaiolli L. Vanzella P. Biazoli C. (2017). Imaging brain function with functional near-infrared spectroscopy in unconstrained environments. *Front. Hum. Neurosci.* 11:258. 10.3389/fnhum.2017.00258 28567011 PMC5434677

[B6] Barker J. W. Aarabi A. Huppert T. J. (2013). Autoregressive model based algorithm for correcting motion and serially correlated errors in fNIRS. *Biomed. Opt. Express* 4:1366. 10.1364/BOE.4.001366 24009999 PMC3756568

[B7] Beauchamp M. S. Argall B. D. Bodurka J. Duyn J. H. Martin A. (2004). Unraveling multisensory integration: patchy organization within human STS multisensory cortex. *Nat. Neurosci.* 7 1190–1192. 10.1038/nn1333 15475952

[B8] Beauchamp M. S. Yasar N. E. Frye R. E. Ro T. (2008). Touch, sound and vision in human superior temporal sulcus. *Neuroimage* 41 1011–1020. 10.1016/j.neuroimage.2008.03.015 18440831 PMC2409200

[B9] Bekkering H. De Bruijn E. R. A. Cuijpers R. H. Newman-Norlund R. Van Schie H. T. Meulenbroek R. (2009). Joint action: neurocognitive mechanisms supporting human interaction. *Top. Cogn. Sci.* 1 340–352. 10.1111/j.1756-8765.2009.01023.x 25164937

[B10] Bernstein M. Yovel G. (2015). Two neural pathways of face processing: a critical evaluation of current models. *Neurosci. Biobehav. Rev.* 55 536–546. 10.1016/j.neubiorev.2015.06.010 26067903

[B11] Bishop L. Goebl W. (2018). Beating time: how ensemble musicians’ cueing gestures communicate beat position and tempo. *Psychol. Music* 46 84–106. 10.1177/0305735617702971 29276332 PMC5718341

[B12] Calvo-Merino B. Glaser D. E. Grèzes J. Passingham R. E. Haggard P. (2005). Action observation and acquired motor skills: an fMRI study with expert dancers. *Cereb. Cortex* 15 1243–1249. 10.1093/cercor/bhi007 15616133

[B13] Calvo-Merino B. Grèzes J. Glaser D. E. Passingham R. E. Haggard P. (2006). Seeing or doing? Influence of visual and motor familiarity in action observation. *Curr. Biol.* 16 1905–1910. 10.1016/j.cub.2006.07.065 17027486

[B14] Carter R. M. Huettel S. A. (2013). A nexus model of the temporal-parietal junction. *Trends Cogn. Sci.* 17 328–336. 10.1016/j.tics.2013.05.007 23790322 PMC3750983

[B15] Chang A. Livingstone S. R. Bosnyak D. J. Trainor L. J. (2017). Body sway reflects leadership in joint music performance. *Proc. Natl. Acad. Sci. U.S.A.* 114 E4134–E4141. 10.1073/pnas.1617657114 28484007 PMC5448222

[B16] Chauvigné L. A. S. Belyk M. Brown S. (2018). Taking two to tango: FMRI analysis of improvised joint action with physical contact. *PLoS One* 13:e0191098. 10.1371/journal.pone.0191098 29324862 PMC5764359

[B17] Cui X. Bryant D. M. Reiss A. L. (2012). NIRS-based hyperscanning reveals increased interpersonal coherence in superior frontal cortex during cooperation. *Neuroimage* 59 2430–2437. 10.1016/j.neuroimage.2011.09.003 21933717 PMC3254802

[B18] D’Ausilio A. Novembre G. Fadiga L. Keller P. E. (2015). What can music tell us about social interaction? *Trends Cogn. Sci.* 19 111–114. 10.1016/j.tics.2015.01.005 25641075

[B19] Decety J. Grèzes J. (2006). The power of simulation: imagining one’s own and other’s behavior. *Brain Res.* 1079 4–14. 10.1016/j.brainres.2005.12.115 16460715

[B20] Delpy D. T. Cope M. (1997). Quantification in tissue near-infrared spectroscopy. *Philos. Trans. R. Soc. B Biol. Sci.* 352 649–659. 10.1098/rstb.1997.0046

[B21] Fairhurst M. T. Janata P. Keller P. E. (2014). Leading the follower: an fMRI investigation of dynamic cooperativity and leader-follower strategies in synchronization with an adaptive virtual partner. *Neuroimage* 84 688–697. 10.1016/j.neuroimage.2013.09.027 24064075

[B22] Gallagher H. L. Frith C. D. (2003). Functional imaging of ‘theory of mind. *Trends Cogn. Sci.* 7 77–83. 10.1016/S1364-6613(02)00025-6 12584026

[B23] Gilaie-Dotan S. Kanai R. Bahrami B. Rees G. Saygin A. P. (2013). Neuroanatomical correlates of biological motion detection. *Neuropsychologia* 51 457–463. 10.1016/j.neuropsychologia.2012.11.027 23211992 PMC3611598

[B24] Goebl W. Palmer C. (2009). Synchronization of timing and motion among performing musicians. *Music Percept.* 26 427–438. 10.1525/mp.2009.26.5.427

[B25] Grossman E. D. Battelli L. Pascual-Leone A. (2005). Repetitive TMS over posterior STS disrupts perception of biological motion. *Vis. Res.* 45 2847–2853. 10.1016/j.visres.2005.05.027 16039692

[B26] Huppert T. J. (2016). Commentary on the statistical properties of noise and its implication on general linear models in functional near-infrared spectroscopy. *Neurophotonics* 3:010401. 10.1117/1.NPh.3.1.010401 26989756 PMC4773699

[B27] Keller P. E. Novembre G. Hove M. J. (2014). Rhythm in joint action: psychological and neurophysiological mechanisms for real-time interpersonal coordination. *Philos. Trans. R. Soc. B Biol. Sci.* 369:20130394. 10.1098/rstb.2013.0394 25385772 PMC4240961

[B28] Knoblich G. Sebanz N. (2008). Evolving intentions for social interaction: from entrainment to joint action. *Philos. Trans. R. Soc. B Biol. Sci.* 363 2021–2031. 10.1098/rstb.2008.0006 18292061 PMC2606699

[B29] Kokal I. Gazzola V. Keysers C. (2009). Acting together in and beyond the mirror neuron system. *Neuroimage* 47 2046–2056. 10.1016/j.neuroimage.2009.06.010 19524043

[B30] Konvalinka I. Bauer M. Stahlhut C. Hansen L. K. Roepstorff A. Frith C. D. (2014). Frontal alpha oscillations distinguish leaders from followers: multivariate decoding of mutually interacting brains. *Neuroimage* 94 79–88. 10.1016/j.neuroimage.2014.03.003 24631790

[B31] Konvalinka I. Vuust P. Roepstorff A. Frith C. D. (2010). Follow you, follow me: continuous mutual prediction and adaptation in joint tapping. *Q. J. Exp. Psychol.* 63 2220–2230. 10.1080/17470218.2010.497843 20694920

[B32] Krall S. C. Rottschy C. Oberwelland E. Bzdok D. Fox P. T. Eickhoff S. B. (2015). The role of the right temporoparietal junction in attention and social interaction as revealed by ALE meta-analysis. *Brain Struct. Funct.* 220 587–604. 10.1007/s00429-014-0803-z 24915964 PMC4791048

[B33] Lindenberger U. Li S. C. Gruber W. Müller V. (2009). Brains swinging in concert: cortical phase synchronization while playing guitar. *BMC Neurosci.* 10:22. 10.1186/1471-2202-10-22 19292892 PMC2662862

[B34] Loehr J. D. Kourtis D. Vesper C. Sebanz N. Knoblich G. (2013). Monitoring individual and joint action outcomes in duet music performance. *J. Cogn. Neurosci.* 25 1049–1061. 10.1162/jocn_a_00388 23489144

[B35] Montague P. R. Berns G. S. Cohen J. D. McClure S. M. Pagnoni G. Dhamala M. (2002). Hyperscanning: simultaneous fMRI during linked Social Interactions. *Neuroimage* 16 1159–1164. 10.1006/NIMG.2002.1150 12202103

[B36] Müller V. Sänger J. Lindenberger U. (2013). Intra- and inter-brain synchronization during musical improvisation on the guitar. *PLoS One* 8:e73852. 10.1371/journal.pone.0073852 24040094 PMC3769391

[B37] Murnighan J. K. Conlon D. E. (1991). The dynamics of intense work groups: a study of british string quartets. *Adm. Sci. Q.* 36 165–186. 10.2307/2393352

[B38] Newman-Norlund R. D. Bosga J. Meulenbroek R. G. J. Bekkering H. (2008). Anatomical substrates of cooperative joint-action in a continuous motor task: virtual lifting and balancing. *Neuroimage* 41 169–177. 10.1016/j.neuroimage.2008.02.026 18378467

[B39] Novembre G. Keller P. E. (2014). A conceptual review on action-perception coupling in the musicians brain: what is it good for? *Front. Hum. Neurosci.* 8:603. 10.3389/fnhum.2014.00603 25191246 PMC4139714

[B40] Novembre G. Ticini L. F. Schütz-Bosbach S. Keller P. E. (2012). Distinguishing self and other in joint action. Evidence from a musical paradigm. *Cereb. Cortex* 22 2894–2903. 10.1093/cercor/bhr364 22235034

[B41] Novembre G. Ticini L. F. Schütz-Bosbach S. Keller P. E. (2014). Motor simulation and the coordination of self and other in real-time joint action. *Soc. Cogn. Affect. Neurosci.* 9 1062–1068. 10.1093/scan/nst086 23709353 PMC4127011

[B42] Pecenka N. Keller P. E. (2011). The role of temporal prediction abilities in interpersonal sensorimotor synchronization. *Exp. Brain Res.* 211 505–515. 10.1007/s00221-011-2616-0 21424257

[B43] Pelphrey K. A. Morris J. P. Michelich C. R. Allison T. McCarthy G. (2005). Functional anatomy of biological motion perception in posterior temporal cortex: an fMRI study of eye, mouth and hand movements. *Cereb. Cortex* 15 1866–1876. 10.1093/cercor/bhi064 15746001

[B44] Ragert M. Schroeder T. Keller P. E. (2013). Knowing too little or too much: the effects of familiarity with a co-performer’s part on interpersonal coordination in musical ensembles. *Front. Psychol.* 4:368. 10.3389/fpsyg.2013.00368 23805116 PMC3691551

[B45] Rizzolatti G. Sinigaglia C. (2010). The functional role of the parieto-frontal mirror circuit: interpretations and misinterpretations. *Nat. Rev. Neurosci.* 11 264–274. 10.1038/nrn2805 20216547

[B46] Sänger J. Müller V. Lindenberger U. (2012). Intra- and interbrain synchronization and network properties when playing guitar in duets. *Front. Hum. Neurosci.* 6:312. 10.3389/fnhum.2012.00312 23226120 PMC3509332

[B47] Sänger J. Müller V. Lindenberger U. (2013). Directionality in hyperbrain networks discriminates between leaders and followers in guitar duets. *Front. Hum. Neurosci.* 7:234. 10.3389/fnhum.2013.00234 23761745 PMC3671173

[B48] Santosa H. Hong M. J. Hong K. S. (2014). Lateralization of music processing with noises in the auditory cortex: an fNIRS study. *Front. Behav. Neurosci.* 8:418. 10.3389/fnbeh.2014.00418 25538583 PMC4260509

[B49] Santosa H. Zhai X. Fishburn F. Huppert T. (2018). The NIRS brain AnalyzIR toolbox. *Algorithms* 11:73 10.3390/a11050073 PMC11218834 38957522

[B50] Saygin A. P. (2007). Superior temporal and premotor brain areas necessary for biological motion perception. *Brain* 130 2452–2461. 10.1093/brain/awm162 17660183

[B51] Scholkmann F. Holper L. Wolf U. Wolf M. (2013). A new methodical approach in neuroscience: assessing inter-personal brain coupling using functional near-infrared imaging (fNIRI) hyperscanning. *Front. Hum. Neurosci.* 7:813. 10.3389/fnhum.2013.00813 24348362 PMC3841755

[B52] Schurz M. Radua J. Aichhorn M. Richlan F. Perner J. (2014). Fractionating theory of mind: a meta-analysis of functional brain imaging studies. *Neurosci. Biobehav. Rev.* 42 9–34. 10.1016/j.neubiorev.2014.01.009 24486722

[B53] Schütz-Bosbach S. Mancini B. Aglioti S. M. Haggard P. (2006). Self and other in the human motor system. *Curr. Biol.* 16 1830–1834. 10.1016/j.cub.2006.07.048 16979561

[B54] Sebanz N. Bekkering H. Knoblich G. (2006). Joint action: bodies and minds moving together. *Trends Cogn. Sci.* 10 70–76. 10.1016/j.tics.2005.12.009 16406326

[B55] Sebanz N. Knoblich G. (2009). Prediction in Joint Action: what. When, and Where. *Top. Cogn. Sci.* 1 353–367. 10.1111/j.1756-8765.2009.01024.x 25164938

[B56] Sharbrough F. Chatrian G. E. Lesser R. P. Lüders H. Nuwer M. Picton T. W. (1991). American electroencephalographic society guidelines for standard electrode position nomenclature. *J. Clin. Neurophysiol.* 8 200–202. 10.1097/00004691-199104000-00007 2050819

[B57] Strangman G. E. Li Z. Zhang Q. (2013). Depth sensitivity and source-detector separations for near infrared spectroscopy based on the colin27 brain template. *PLoS One* 8:e66319. 10.1371/journal.pone.0066319 23936292 PMC3731322

[B58] Timmers R. Endo S. Bradbury A. Wing A. M. (2014). Synchronization and leadership in string quartet performance: a case study of auditory and visual cues. *Front. Psychol.* 5:645. 10.3389/fpsyg.2014.00645 25002856 PMC4066619

[B59] Tsakiris M. Costantini M. Haggard P. (2008). The role of the right temporo-parietal junction in maintaining a coherent sense of one’s body. *Neuropsychologia* 46 3014–3018. 10.1016/j.neuropsychologia.2008.06.004 18601939

[B60] Van Der Steen M. C. Jacoby N. Fairhurst M. T. Keller P. E. (2015). Sensorimotor synchronization with tempo-changing auditory sequences: modeling temporal adaptation and anticipation. *Brain Res.* 1626 66–87. 10.1016/j.brainres.2015.01.053 25725379

[B61] Van Kemenade B. M. Seymour K. Wacker E. Spitzer B. Blankenburg F. Sterzer P. (2014). Tactile and visual motion direction processing in hMT+/V5. *Neuroimage* 84 420–427. 10.1016/j.neuroimage.2013.09.004 24036354

[B62] Van Overwalle F. (2009). Social cognition and the brain: a meta-analysis. *Hum. Brain Mapp.* 30 829–858. 10.1002/hbm.20547 18381770 PMC6870808

[B63] Vetter P. Grosbras M. H. Muckli L. (2015). TMS over V5 disrupts motion prediction. *Cereb. Cortex* 25 1052–1059. 10.1093/cercor/bht297 24152544 PMC4380002

[B64] Villringer A. Planck J. Hock C. Schleinkofer L. Dirnagl U. (1993). Near infrared spectroscopy (NIRS): a new tool to study hemodynamic changes during activation of brain function in human adults. *Neurosci. Lett.* 154 101–104. 10.1016/0304-3940(93)90181-J 8361619

[B65] Volpe G. D’Ausilio A. Badino L. Camurri A. Fadiga L. (2016). Measuring social interaction in music ensembles. *Philos. Trans. R. Soc. B Biol. Sci.* 371:20150377. 10.1098/rstb.2015.0377 27069054 PMC4843615

[B66] Wing A. M. Endo S. Bradbury A. Vorberg D. (2014). Optimal feedback correction in string quartet synchronization. *J. R. Soc. Interface* 11 20131125–20131125. 10.1098/rsif.2013.1125 24478285 PMC3928944

[B67] Yang D. Y.-J. Rosenblau G. Keifer C. Pelphrey K. A. (2015). An integrative neural model of social perception, action observation, and theory of mind. *Neurosci. Biobehav. Rev.* 51 263–275. 10.1016/j.neubiorev.2015.01.020 25660957 PMC4940188

