# Supplementary figures and images for: fNIRS Responses in Professional Violinists While Playing Duets: Evidence for Distinct Leader and Follower Roles at the Brain Level
Source: Front Psychol. 2019 Feb 5;10:164. doi: 10.3389/fpsyg.2019.00164 (PMC6370678; doi:10.3389/fpsyg.2019.00164)

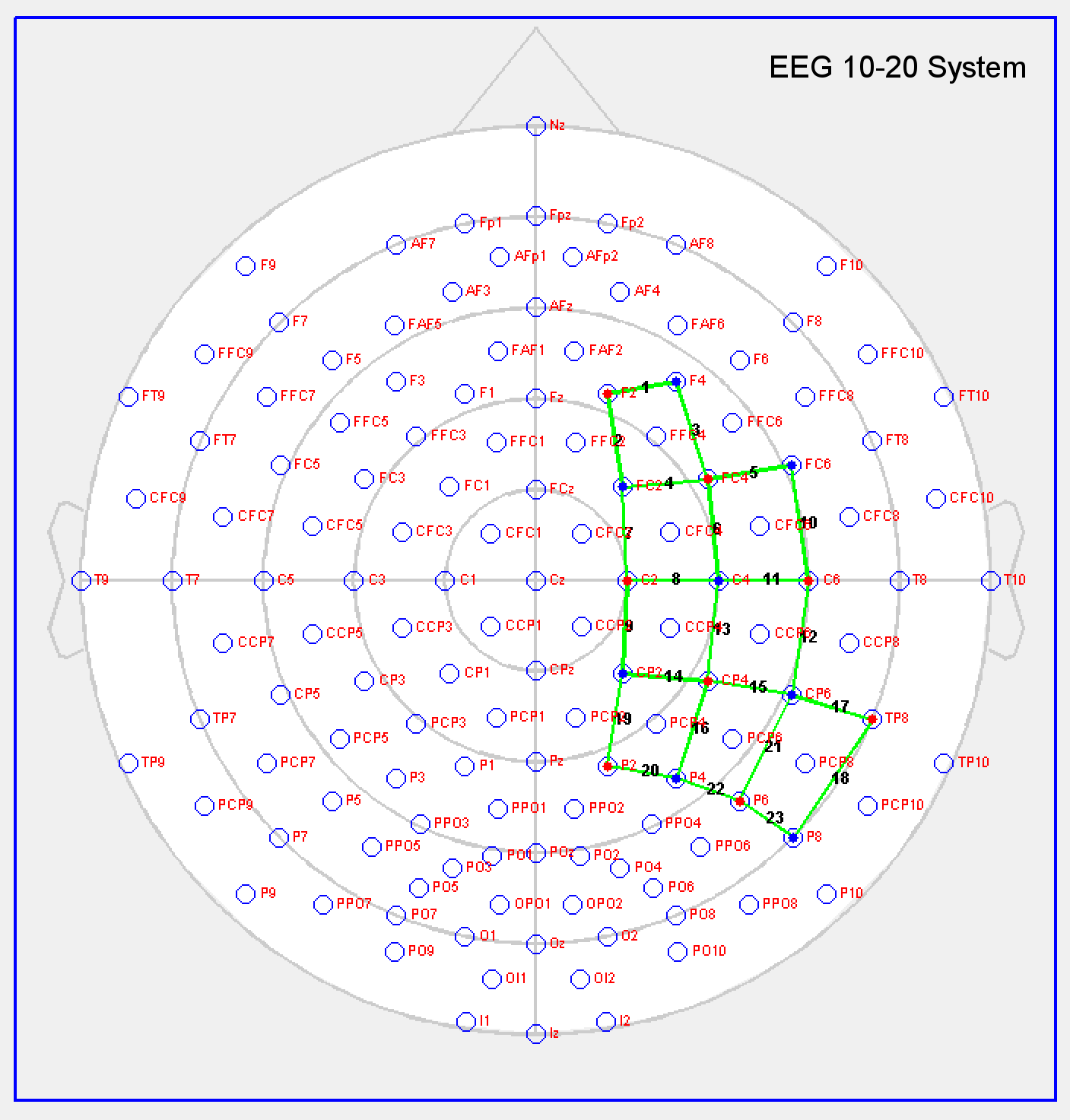

Supplement: Figure S1 — Optode setup overlaid onto the 10–20 international system. Sources are depicted in red, detectors in blue, and channels in green. [file Image_1.TIF]

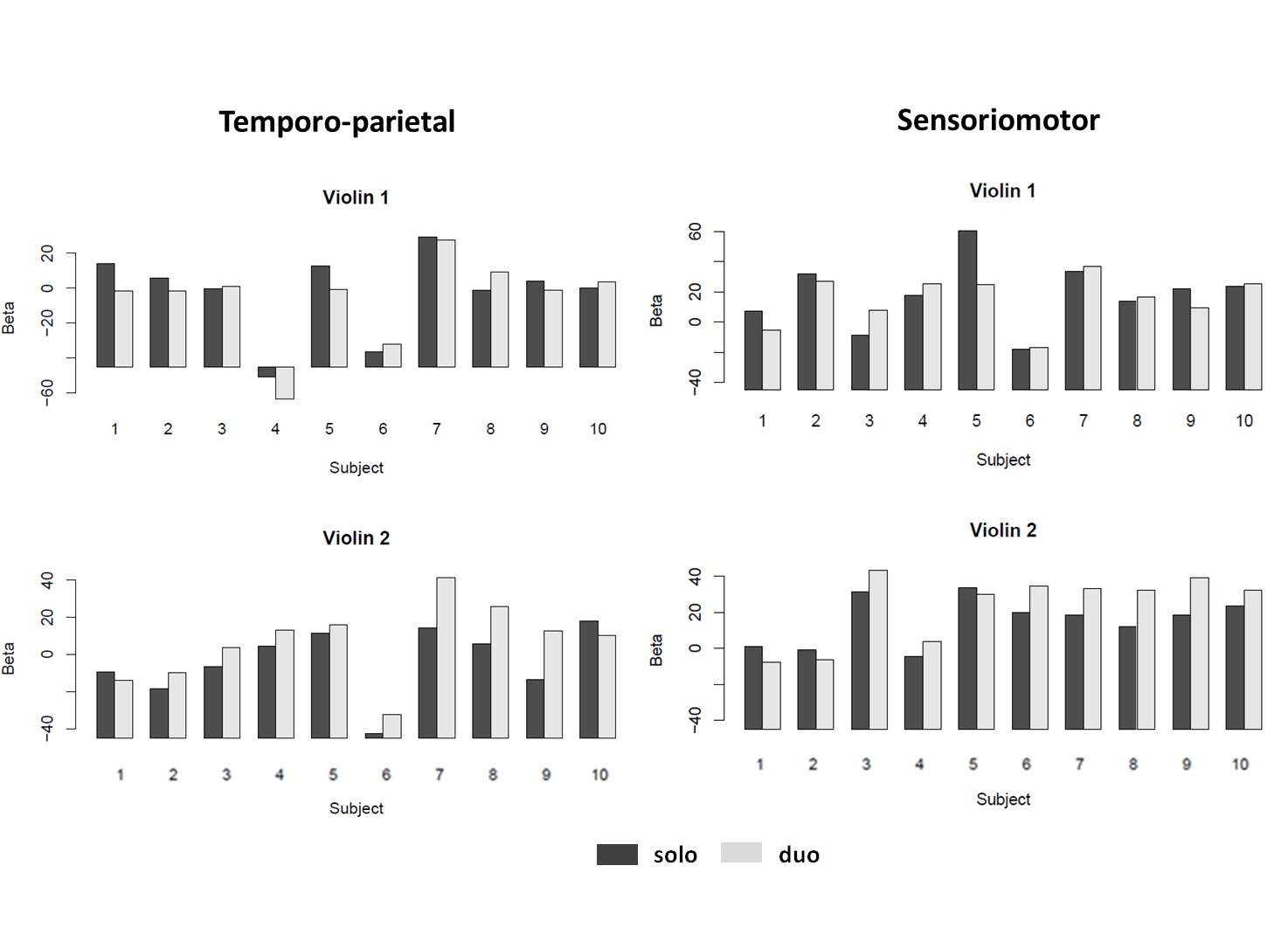

Supplement: Figure S2 — Individual beta values of the group comparison between duo versus solo when playing as Violin 1 and Violin 2. [file Image_2.TIF]
